# Understanding Drug Repurposing From the Perspective of Biomedical Entities and Their Evolution: Bibliographic Research Using Aspirin

**DOI:** 10.2196/16739

**Published:** 2020-06-16

**Authors:** Xin Li, Justin F Rousseau, Ying Ding, Min Song, Wei Lu

**Affiliations:** 1 Information Retrieval and Knowledge Mining Laboratory School of Information Management Wuhan University Wuhan China; 2 School of Informatics, Computing, and Engineering Indiana University Bloomington, IN United States; 3 Department of Population Health and Department of Neurology Dell Medical School The University of Texas at Austin Austin, TX United States; 4 School of Information Dell Medical School The University of Texas Austin Austin, TX United States; 5 Department of Library and Information Science Yonsei University Seoul Republic of Korea

**Keywords:** drug repurposing, biomedical entities, entitymetrics, bibliometrics, aspirin, acetylsalicylic acid

## Abstract

**Background:**

Drug development is still a costly and time-consuming process with a low rate of success. Drug repurposing (DR) has attracted significant attention because of its significant advantages over traditional approaches in terms of development time, cost, and safety. Entitymetrics, defined as bibliometric indicators based on biomedical entities (eg, diseases, drugs, and genes) studied in the biomedical literature, make it possible for researchers to measure knowledge evolution and the transfer of drug research.

**Objective:**

The purpose of this study was to understand DR from the perspective of biomedical entities (diseases, drugs, and genes) and their evolution.

**Methods:**

In the work reported in this paper, we extended the bibliometric indicators of biomedical entities mentioned in PubMed to detect potential patterns of biomedical entities in various phases of drug research and investigate the factors driving DR. We used aspirin (acetylsalicylic acid) as the subject of the study since it can be repurposed for many applications. We propose 4 easy, transparent measures based on entitymetrics to investigate DR for aspirin: Popularity Index (P_1_), Promising Index (P_2_), Prestige Index (P_3_), and Collaboration Index (CI).

**Results:**

We found that the maxima of P_1_, P_3_, and CI are closely associated with the different repurposing phases of aspirin. These metrics enabled us to observe the way in which biomedical entities interacted with the drug during the various phases of DR and to analyze the potential driving factors for DR at the entity level. P_1_ and CI were indicative of the dynamic trends of a specific biomedical entity over a long time period, while P_2_ was more sensitive to immediate changes. P_3_ reflected the early signs of the practical value of biomedical entities and could be valuable for tracking the research frontiers of a drug.

**Conclusions:**

In-depth studies of side effects and mechanisms, fierce market competition, and advanced life science technologies are driving factors for DR. This study showcases the way in which researchers can examine the evolution of DR using entitymetrics, an approach that can be valuable for enhancing decision making in the field of drug discovery and development.

## Introduction

### Background

Despite recent advances in life sciences and technology, drug development is still a costly and time-consuming process with a low rate of success [1]. Discovering a new drug usually takes more than 10 years and costs around $2 billion on average [2]. The number of targetable human genes is approximately 3000, and the identification of serious and even deadly drug side effects is ongoing [3,4]. To overcome these difficulties, many researchers have turned to drug repurposing, which is the practice of identifying novel clinical indicators for existing marketed drugs [5-7].

The past few decades have produced a few successful cases of drug repurposing. For example, sildenafil, originally developed to treat cardiovascular disease, was unexpectedly discovered to be effective against erectile dysfunction [8]. Thalidomide, once used for morning sickness, has been repurposed for the treatment of multiple myeloma [9], and metformin, originally a treatment for type 2 diabetes, has been studied for the treatment of depression, aging, obesity, and even cancer [10,11]. Beta blockers, initially indicated for hypertension, and topiramate, originally used as an antiepileptic, are both repurposed for migraineurs [12,13]. Because of its significant advantages over traditional approaches, in terms of development time, cost, and previous clinical studies, drug repurposing has attracted significant attention from pharmaceutical firms, scientists, and governments in recent years [7,14].

Methodologies for drug repurposing and their successful applications have been widely discussed. Chen et al [15] designed a system-based algorithm called the reverse gene expression score based on several large-scale publicly accessible datasets and demonstrated the potency and efficacy of vorinostat, geldanamycin, and gemcitabine for the treatment of liver cancers. Xu et al [16] found that emricasan had an inhibitory effect on the Zika virus by screening more than 6000 compounds. With the rapid development of natural language processing and deep learning techniques, robust solutions have recently been proposed and have demonstrated potential. Researchers have integrated more than 20 different datasets into a knowledge graph to predict potential drug and target pairs [17-19]. Hamilton et al [20] queried drug-gene-drug interactions within a low-dimensional embedding of biomedical knowledge graphs to predict missing or unobserved links for drug repurposing. Chang et al [21] proposed a novel deep learning model called “CDRscan” that can successfully predict the feasibility of drug repurposing and recommend the most effective anticancer agents for an individual patient. Öztürk et al [22] represented drugs and protein sequences using convolutional neural networks to predict the binding affinities of drug-target interactions.

Academic publications are produced at high volume, with around 3000 new articles currently published per day [23]. No researcher nor clinician can read and comprehend all the relevant articles in their domain [24]. The “known” knowledge has turned into “unknown known” knowledge, with hidden information and patterns waiting to be discovered. This growing body of scholarly data opens a new era of exploiting literature and data to enable data-driven discovery [24]. Literature-based discovery, which connects disconnected entities in literature in PubMed, has been successful in identifying several cases of drug repurposing, such as fish oil for Raynaud’s syndrome, magnesium for migraine headaches, and proton pump inhibitors for atrial fibrillation [25-27]. Swanson [26] demonstrated that bibliometrics can be a useful approach to knowledge discovery and recommended that his method could be extended to other disconnected sets of scientific literature to enable cross-disciplinary innovation [28]. With entitymetrics — bibliometric indicators based on entities studied in the medical literature — researchers without domain knowledge can understand the medical function of a drug [29], identify complex undiscovered biological relationships between drugs and targets [30], and detect implicit gene-gene relationships using literature in PubMed [31]. This research demonstrates the potential of applying bibliometrics to medicine to support data-driven discovery. It represents the next generation of bibliometric studies [32] and already shows great promise [33].

### Objectives

In this research, we extended bibliometric indicators for biomedical entities mentioned in the PubMed literature to investigate drug repurposing. We used aspirin (salicylic acid) as the target drug. Aspirin is one of the most well-recognized and well-studied drugs with a history dating back to 1500 BC [34]. It was originally used as an analgesic to treat mild to moderate pain. It has been used clinically for the treatment of at least 10 diseases, including coronary artery disease, cerebrovascular disease, peripheral arterial disease, preeclampsia, diabetes, colorectal cancer, Kawasaki disease, Alzheimer’s disease, and arthritis [34,35]. New indications for aspirin are still being reported [36-38]. Aspirin has a remarkably wide range of effects and therefore provides an ideal case with which to study drug repurposing. The work described in this paper primarily aimed to identify patterns in the different repurposing phases of aspirin by analyzing the diseases, drugs, and genes related to aspirin. We propose 4 measures based on entitymetrics to identify the characteristics and patterns of drug repurposing for aspirin: Popularity Index (P_1_), Promising Index (P_2_), Prestige Index (P_3_), and Collaboration Index (CI).

### Related Work

#### Drug Repurposing

Drug repurposing has become a dynamic emerging field of drug discovery and development. According to Baker et al [39], in 2018 nearly two-thirds of 35,000 drugs or compounds described in MEDLINE were investigated as potential treatments for diseases other than those for which they were originally indicated. Nearly 200 drugs have been investigated for repurposing for more than 300 diseases. Many successfully repurposed drugs were discovered accidentally, such as the application of thalidomide for multiple myeloma [9] and sildenafil for erectile dysfunction [8].

Approaches have been proposed for the generation of hypotheses about novel drug-target interactions and have been used to develop promising directions for subsequent validation of drug repurposing. In polypharmacology, researchers have proposed 2 types of hypotheses: (1) two drugs could be indicated for the same condition when they produce a similar gene expression profile, and (2) a disease could be one of the indications for a given drug when it has an opposite gene expression profile to that produced by the drug. The Connectivity Map (CMap; Broad Institute, Cambridge, MA), a database for more than 7000 gene-expression profiles of 1309 compounds, has been widely used in this context in previous work. Using a systematic analysis tool, L1000FWD [40], and CMap, Liu et al [41] found that the anticancer drugs KM-00927 and BRD-K75081836 can be used to inhibit histone deacetylase. Kidnapillai et al [42] used gene expression signature data and CMap to identify 10 drugs, including camptothecin, nimesulide, and rescinnamine, that could be effective against bipolar disorder.

In the field of genetics, association analysis has been extensively applied to the interactions between drug targets and diseases to increase the efficiency of drug repurposing. One of the most successful cases in the field of drug repurposing was based on a genome-wide association study (GWAS) [43]. Using GWAS-driven methods, Sanseau et al [44] concluded that 15.6% of genes are the targets of marketed drugs. They found that GWAS traits can be matched with the indications of drugs and genes involved in pathogenesis have a high probability of being targets for drug repurposing. Based on a strong association between the gene TNFSF11 and Crohn’s disease, the authors inferred, and subsequently confirmed, that dishubzumab, originally developed for the treatment of osteoporosis, can be used against Crohn’s disease [44]. Ferrero and Agarwal [45] combined a CMap-based approach with perturbation of transcriptional profiles and disease data from GWAS for target prioritization and drug repurposing. These researchers pointed out that genetic evidence is important in maximizing the success rate of drug repurposing.

These methods in polypharmacology and genetics usually rely on the high-throughput screening of massive amounts of data related to compounds and targets. As knowledge about drug targets accumulates and computational chemistry rapidly develops, simulations of the interactions between drugs and proteins have shown the potential to replace traditional high-throughput screening. Dakshanamurthy et al [46] proposed a proteochemometric method called “train, match, fit, streamline” to conduct molecular docking of over 3000 FDA-approved compounds across the crystal structures of more than 2000 human targets. They found that mebendazole could be used for the inhibition of VEGFR2 kinase and that celecoxib was a promising therapy for malignancies because it binds an adhesion molecule, cadherin-11. Li et al [47] designed a standalone approach to dock over 30 crystal structures of MAPK_14_ and BIM-8 with all drugs from DrugBank and found that nilotinib, as a potential inhibitor of MAPK_14_, could be a cure for inflammatory diseases.

Another significant source of drug repurposing is drug side effects. Typical instances of side effect–based drug repurposing include the use of sildenafil for erectile dysfunction [8] and the application of exenatide acetate for obesity [48], both of which were “happy accidents.” Recently, Yang and Agarwal [49] generated human phenotypic profiles for drugs based on over 3000 side-effect relationships extracted from PharmGKB and employed naïve Bayes methods to identify new indications for drugs according to their side effects. This study also suggested that the use of side effects is a type of clinical phenotypic assay and side effects should be rationally investigated to predict repurposing opportunities for drugs. Ye et al [50] contend that drugs with similar side effects could share the same indications because they may have the same or similar mechanisms of action. Using a side effect similarity–based drug-drug network, they transformed drug repurposing into an information retrieval issue and successfully obtained the top 5 indications of 1234 drugs approved by the FDA.

With the rise of machine learning and deep learning in computer science and bioinformatics, the problem of drug repurposing has been addressed using approaches such as classification [51,52], link prediction [53,54], entity prediction [53], and path prediction [18,55]. Liang et al [53] represented biomedical entities and their relationships in a heterogeneous network using graph2vec and knowledge2vec [56] and employed a cascade learning model to find potential interactions between drugs, genes, diseases, and treatments. They found that vitamin D could be a treatment for prostate cancer. Fu et al [55] treated drug repurposing as a binary classification problem and combined the metapath-based topological features of biomedical entities in Chem2Bio2RDF and a supervised machine learning model to predict links between drugs and targets. They found that the intrinsic feature selection Random Forest algorithm can be valuable for selecting significant topological features for the prediction of links between drugs and genes.

#### Big Scholarly Data for Medical Knowledge Discovery

Traditionally, knowledge discovery in medical domains has relied on first-hand observation such as epidemiological statistics, follow-ups, and laboratory-generated experimental data [24]. A large number of research papers are published daily, posing significant challenges for scientists wishing to have a comprehensive understanding of their domain [24]. The “known” knowledge has turned into “undiscovered public knowledge,” with patterns and information waiting to be uncovered. This large body of literature and data also provides rich opportunities for researchers to undertake data-driven knowledge discovery. The usefulness of literature-based discovery has been demonstrated in many previous research projects. For instance, the “ABC” model proposed by Swanson in 1986 [25] was used to discover relationships between biomedical entities, such as Raynaud’s syndrome and fish oil [25], migraine headaches and magnesium [26], and atrial fibrillation and proton pump inhibitors [27]. The “ABC” model is co-occurrence–based and is based upon the premise that seemingly unrelated concepts A and C could be related when there is a concept B related to both A and C [27]. Since Swanson’s research, various modifications of the “ABC” model have been proposed to discover hidden relationships among biomedical concepts in PubMed, such as ontology-based entity mapping [57], network-based entity extraction [58], and semantic path–based storytelling [59]. The “ABC” model and its variants indicate that bibliometrics can be a valuable method for medical knowledge discovery in the era of big scholarly data.

Knowledge graphs of big scholarly data can contain nodes representing biomedical entities such as diseases, drugs, genes, pathways, and cell lines and non-biomedical entities such as authors, institutions, articles, journals, conferences, and keywords. Edges in the graph can represent the relationships between the biomedical entities in the literature. Lv et al [60] established a therapeutic knowledge graph for autism using drug entities and MeSH terms extracted from about 20,000 articles relating to autism published between 1946 and 2015. They proposed a novel topology-driven method incorporating various graph-analytical techniques for drug discovery and concluded that tocilizumab, sulfisoxazole, tacrolimus, and prednisone were promising for the treatment of autism. Ding et al [29] constructed an entity-entity citation graph to highlight the significance of biomedical entities embedded in literature for future knowledge discovery. Researchers have also integrated big scholarly data with other publicly accessible biomedical datasets, such as DrugBank [61], Gene Ontology [62], and SIDER [63], to form a comprehensive knowledge graph for medical knowledge discovery. A typical example is the Chem2Bio2RDF database, created by integrating more than 20 chemogenomic datasets with PubMed. Wang et al [30] proposed a novel algorithm called Bio-LDA to automatically extract latent topics in life sciences and identified relationships and patterns among compounds, genes, and diseases from Chem2Bio2RDF. He et al [64] designed a graph-mining algorithm to predict potential relationships between different biomedical entities. The case they studied demonstrated that the antidiabetic drug rosiglitazone has cardiovascular-related side effects.

Entitymetrics, an entity-driven bibliometric method, and the next generation of citation analysis [29,32] make it possible for researchers without domain knowledge to measure the impact, usage, and transfer of knowledge entities embedded in the academic literature for further knowledge discovery [32]. Ding et al [29] built an entity-entity citation graph based on articles related to metformin and detected most of the known interactions of metformin with biomedical entities. Williams et al [65] recognized and quantified relationships between academic discoveries and major advances in the domain of two new drugs, ipilimumab and ivacaftor, to enhance government support and public understanding. Zhu et al [66] established paper-entity, entity-entity co-occurrence, and entity-specific networks based on the scientific literature to investigate the evolution of hepatic carcinoma at a granular level. Lv et al [60] discovered new indications for drugs using topology-driven trend analysis of drug-drug and drug-indication networks. These studies demonstrate the potential of the application of bibliometric methods to data-driven discovery in medical domains.

Drug repurposing, as one of the most significant issues in the field of medical knowledge discovery, has been extensively investigated [17,23,24,27,28,55-57,64]. In this research, we extended the bibliometric indicators for biomedical entities described in the PubMed literature to understand the process of drug repurposing.

## Methods

### Data Collection

Papers on aspirin-related research published between 1951 and 2018 were collected from PubMed. Since aspirin is known by many names, the search terms were chosen from DrugBank, RxNorm, and MeSH terms [33,61]. The final search query is shown in Textbox 1. Non-journal articles, non-English articles, letters, and editorial commentaries were excluded. In total, 63,387 publications from PubMed were downloaded in XML format.

**Figure 1:**
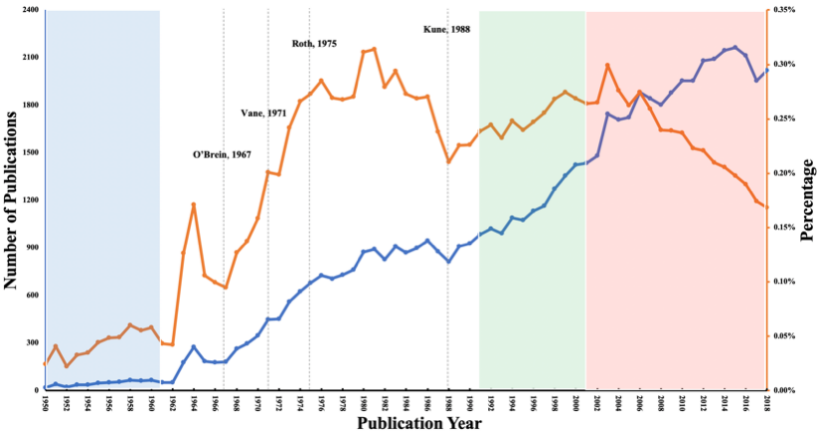
Number of aspirin-related studies in PubMed over time. The background colors indicate the 4 phases of aspirin research.

To better understand the drug repurposing process of aspirin, the relevant research was divided into 4 phases based on previous studies [34,35] and expert suggestions: (1) 1951-1960, the original use; (2) 1961-1990, in-depth studies of pharmacological mechanisms and side effects; (3) 1991-2000, repurposing for cardiovascular diseases; and (4) 2001-2018, repurposing for other diseases, such as colorectal cancer and breast cancer. These phases can also be observed from the evolution and trends of the publications, as shown in Figure 1 and Table 1.

Before extracting biomedical entities, all articles were parsed to obtain PMIDs, publication years, titles, abstracts, authors, journals, and institutions using a dom4j XML parser written in Java. Then, we used spaCy for preprocessing (such as removing the punctuation and stop words) of titles and abstracts in the natural language processing pipeline. In addition, a novel and reliable method of author name disambiguation proposed by Lerchenmueller and Sorenson [67] was used to count distinct authors.

**Search query used for retrieving aspirin-related publications.**

(((aspirin) OR ( acetylsalicylic acid) OR (acid, acetylsalicylic) OR (“2-(acetyloxy)benzoic aci”) OR (acylpyrin) OR (aloxiprimum) OR (colfarit) OR (dispril) OR (easprin) OR (ecotrin) OR (endosprin) OR (magnecyl) OR (micristin) OR (polopiri) OR (polopiryna) OR (solprin) OR (solupsan) OR (zorprin) OR (acetysal) OR (2-acetoxybenzenecarboxylic acid) OR (2-acetoxybenzoic acid) OR (acetylsalicylate) OR (acetylsalicylsäure) OR (“acide 2-(acétyloxy)benzoïqu”) OR (acide acétylsalicylique) OR (ácido acetilsalicílico) OR (acidum acetylsalicylicum) OR (aspirina) OR (azetylsalizylsäure) OR (o-acetoxybenzoic acid) OR (o-acetylsalicylic acid) OR (o-carboxyphenyl acetate) OR (salicylic acid acetate) ) AND (“1951”[PDAT] : “2018”[PDAT]))

**Table 1:** Descriptive statistics of the 4 phases of aspirin research.

Phases, Time span		Number of publications		Number of authors		Average number of authors		Number of journals	
**1. Original use**									
	1951-1955	208		318		1.76		117	
	1956-1960	299		498		1.88		159	
	1951-1960	507		794		1.83		218	
**2. In-depth studies of pharmacological mechanisms and side effects**									
	1961-1965	748		1310		2.01		301	
	1966-1970	1268		2167		2.12		418	
	1971-1975	2766		4880		2.40		696	
	1976-1980	3797		7419		2.71		895	
	1981-1985	4395		10,011		3.16		1033	
	1986-1990	4470		11,600		3.50		1101	
	1961-1990	17,444		31,787		2.90		2153	
**3. Repurposing for cardiovascular diseases**									
	1991-1995	5164		14,044		3.69		1256	
	1996-2000	6353		17,694		4.10		1314	
	1991-2000	11,517		28,818		3.91		1798	
**4. Repurposing for other diseases**									
	2001-2005	8099		27,784		4.22		1719	
	2006-2010	9366		35,313		4.94		1974	
	2011-2015	10,436		44,603		5.78		2410	
	2016-2018	6018		30,796		6.73		1881	
	2001-2018	33,919		118,857		5.33		3865	
Total			63,387		171,559		4.39		5443

### Biomedical Entity Extraction

The biomedical entity extraction module provided by the biomedical entity search tool (BEST) [68], a dictionary-based biomedical information extraction tool based on sophisticated information retrieval approaches, was deployed to extract entities such as diseases, drugs, and genes. The BEST dictionary is built from 12 different public sources, including NCBI Entrez Gene, DrugBank, T3DB, Animal TFDB, Therapeutic Target DataBase, PubChem, and MeSH [68]. We obtained 1472 unique disease names, 1640 unique drug names, and 3184 unique gene names from the titles and abstracts. Table 2 shows the top 10 biomedical entities of 3 different types and their frequency of appearance in PubMed articles.

**Table 2:** Top 10 biomedical entities in aspirin-related publications during 1951-2018.

Rank	Diseases	Frequency of diseases	Drugs	Frequency of drugs	Genes	Frequency of genes
1	Coronary disease	2707	Clopidogrel	6223	COX-2	3957
2	Asthma	2277	Ticlopidine	5433	CD143	1495
3	Diabetes	1840	Heparin	4391	COX-1	1179
4	Hypersensitivities, drug	1342	Indomethacin	3462	Plasminogen	1131
5	Ulcer, gastric	1146	Warfarin	3457	LDLCQ3	1081
6	Cerebral ischemia	1135	Vitamin F	2760	LPLA2	1047
7	Intracranial vascular disorder	1133	Dipyridamole	2232	GPIIb	1017
8	Ischemic heart disease	1090	Adenosine	2188	P2Y_12_	855
9	Carcinomas, colorectal	1085	Acetaminophen	2099	tPA	748
10	Rheumatoid arthritis	832	Prostacyclin	1498	TNF-α	629

### Entitymetric Indicators for Biomedical Entities (P3C)

In order to quantify and visualize the academic importance of individual biomedical entities, 4 transparent and easy entitymetric indexes (P3C) were developed: Popularity Index (P_1_), Promising Index (P_2_), Prestige Index (P_3_), and Collaboration Index (CI). These indicators can be considered as the extensions of the indicators proposed by Kissin and Edwin [33] and Kissin [69] for measuring the academic interest of a drug or technique at the article level. In this study, we adapted the indicators from the perspective of biomedical entities with the goal of understanding drug repurposing. Different from Kissin’s indicators, our indicators not only focus on the articles on a given drug but also consider the changes in indicators of biomedical entities (eg, diseases, drugs, and genes) and non-biomedical entities (eg, authors) that are related to the given drug. Detailed explanations of these measures are provided in the following sections.

#### Popularity Index (P_1_)

The P_1_ of a certain biomedical entity reflects the percentage of publications discussing that biomedical entity among all publications in a research field during a specific period, usually 5 years. The popularity of a biomedical entity *i*, P_1_ (i), is given by:

P_1_ (i) = (N_i_ / N_T_) * 100% (**1**)

where N_i_ is the number of publications relating to an entity *i* in a period, and N_T_ represents the total number of publications in the research field during the same period. An increase in P_1_ indicates growing academic interest in *i* in the field.

#### Promising Index (P_2_)

The P_2_ of a biomedical entity is the change in the popularity of an entity *i* in a research field between two continuous periods. The promising index of a specific biomedical entity *i*, P_2_ (i), is expressed as:

P_2_ (i) = (N_i_ / N_T_) – (N_pi_ / N_pT_) (**2**)

where (N_pi_ / N_pT_) refers to the popularity of the entity *i* in the research field during a previous period of the same length as N_i_. P_2_ reflects the change in the academic interest in a biomedical entity in a research field in two time periods. When P_2_ (i) > 0, it means the academic interest in *i* has increased and vice versa.

#### Prestige Index (P_3_)

P_3_ is defined as the ratio of the number of publications about a specific biomedical entity published in the top journals compared to the number of publications about the same entity in all journals that were indexed by PubMed during the same time period. The prestige of a biomedical entity *i*, P_3_ (i), is calculated as:

P_3_ (i) = (N_H20_ / N_i_) * 100% (**3**)

where N_H20_ represents the number of publications on *i* in the top 20 journals during the same period as N_i_. In this study, the top 20 journals were selected based on the journal impact factor and specialty areas. These journals can be divided into two categories: multidisciplinary journals and specialty journals. Fourteen multidisciplinary journals, including JAMA, The Lancet, BMJ, and similar publications, are common for all diseases, drugs, and genes that were studied in this paper. The other 6 journals, such as Circulation, Blood, and The European Heart Journal, are highly associated with aspirin-related specialty areas. The full list of the top 20 journals is shown in Multimedia Appendix 1. P_3_ reflects the potential significance of a specific biomedical entity. Continuing high prestige scores could be an early signal of the success of entity-related drug discovery or repurposing [69]. We employed a threshold of 5% to indicate that P_3_ was of interest [69].

#### Collaboration Index (CI)

The CI of a biomedical entity reflects the percentage of the number of distinct authors of articles discussing this entity out of all the distinct authors in the research domain over a period of time. The CI of a biomedical entity *i*, CI (i), is calculated by:

CI (i) = (N_AI_ / N_AT_) * 100% (**4**)

where N_AI_ is the number of distinct authors of the publications relating to *i* in a period, and N_AT_ represents the total number of distinct authors in the field in the same period. The CI reflects the research strength of entity *i* in a research field, and a threshold of 5% indicates a level of interest [69].

## Results

### Overview of Aspirin-Related Studies

Figure 1 shows an overview of aspirin-related research in PubMed from 1951 to 2018. The red and blue lines represent the percentage and absolute numbers, respectively, of articles in PubMed per year. The details of the publications, authors, and journals are shown in Table 1. During the evolution of aspirin, Phase 1 (1951-1960) produced 507 articles, most of which were published in journals covering pharmacy-related or general medicine–related topics (Table 1 and Multimedia Appendix 2). Research in Phase I focused on the anti-inflammatory and antipyretic uses of aspirin, and this phase marks the original use of aspirin.

In Phase 2 (1961-1990), a turning point can be identified in 1967, after which the number of relevant papers per year grew dramatically until 1986. Several significant pharmacological discoveries related to aspirin occurred during this period, including the antiplatelet effect [70], mechanism of inhibition of prostaglandin synthesis [71], and acetylation of the cyclo-oxygenase enzyme [72]. The percentage of aspirin-related articles in PubMed reached its peak in 1981, at about 0.32%, and then decreased. Kune et al [73] reported that aspirin could effectively reduce the incidence of colorectal cancer, after which the percentage began to rise again. After 1975, articles began to occur frequently in journals covering specialty areas, such as Circulation and Thrombosis Research. We identify this phase as the in-depth investigation of the pharmacological mechanisms and side effects of aspirin.

In Phase 3 (1991-2000), there was a steady and stable growth in the number and percentage of aspirin-related articles per year in PubMed (Figure 1). Compared to the first 10 years (1951-1960), there was a >22-fold increase in the number of articles as well as a >36-fold increase in the number of distinct authors. As shown in Multimedia Appendix 2, in both 1991-1995 and 1996-2000, 4 of the top 5 journals were cardiovascular-related journals. We thus identify this phase as repurposing for cardiovascular diseases.

In Phase 4 (2001-2018), the number of articles per year grew continuously and reached its peak (2164) in 2015, but the percentage significantly reduced (Figure 1). From the information presented in Table 1, we note that the numbers of articles, distinct authors, and journals in Phase 4 were all higher than those in the previous 3 periods. The average number of authors in this period had exceeded the total average (4.39). Journals covering other topics, for example Cancer Management and Research, Drugs & Aging, and World Neurosurgery, were increasingly represented (Multimedia Appendix 2), demonstrating that aspirin had been experimentally applied to many other diseases. We thus mark this phase as repurposing for other diseases.

To analyze drug repurposing through all 4 phases from the biomedical entity perspective, we first computed the P3C indicators of the top 10 diseases, drugs, and genes in the cohort of aspirin articles during the period 1951-2018. The results show that there are distinct patterns of these indicators in different repurposing phases. To describe these patterns in detail, we reorganized the 30 biomedical entities (the top 10 diseases, top 10 drugs, and top 10 genes) into the 4 phases of aspirin research, according to when each achieved its maximum P_1_, which indicates the focus of research in the field of aspirin. In each phase, we further analyzed the change patterns of the P3C indicators for the most popular biomedical entities, to investigate the features of different phases of drug repurposing, association between entities and P3C indicators, and possible factors driving drug repurposing at the biomedical entity level.

### Before Repurposing

**Figure 2:**
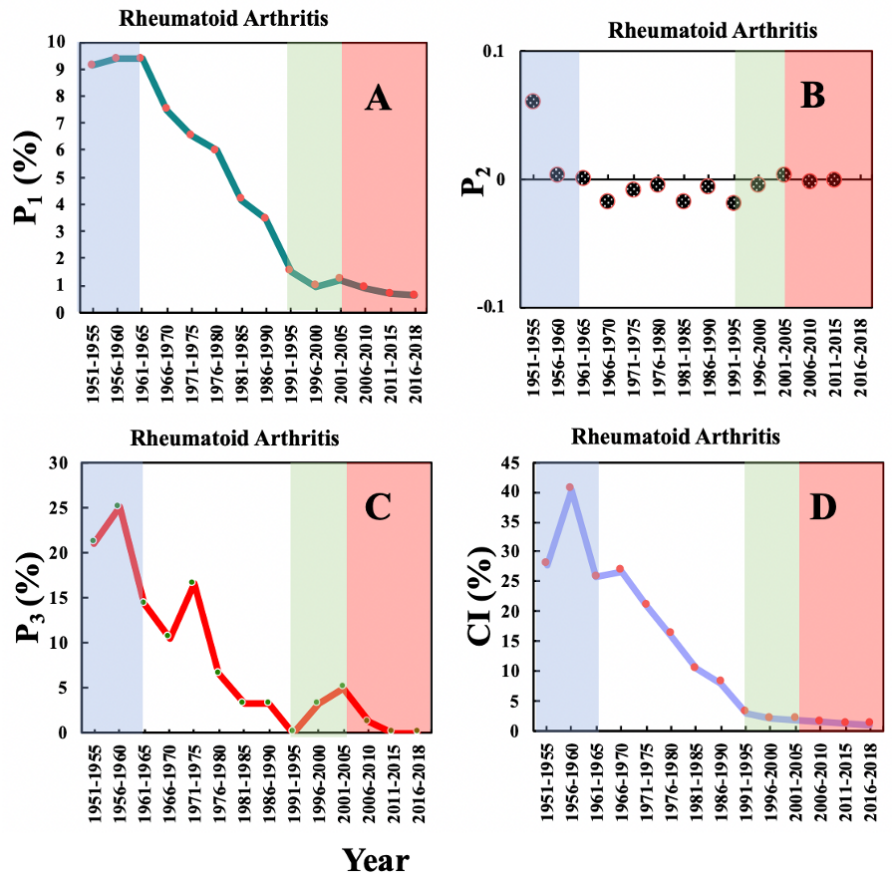
The 4 entitymetric indexes of the biomedical entity “Rheumatoid Arthritis” over time. The background colors indicate the 4 phases of aspirin research.

Only “rheumatoid arthritis” (RA) reached its maximum P_1_ in Phase 1, at 9.36%, as shown in Figure 2A and then exhibited a downhill trend for the rest of the 3 phases, reaching a low of 0.63% in 2016-2018. As shown in Figure 2B, for the P_2_ of RA, there is only one significant increase of more than 0 in all 4 phases: 0.06 in 1951-1955 (Phase 1). This observation indicates that the popularity of RA in 1951-1955 increased by 6% compared to that in 1945-1950. It can also be observed from Figure 2C that the P_3_ of RA was more than 5% during 1951-1980 and reached its maximum in Phase 1 (25%, 1960-1965), indicating that one quarter of the papers studying RA were published in the top 20 journals in the aspirin domain in Phase 1. In the next 3 phases, the P_3_ peaked twice, in Phase 2 (1971-1975) and Phase 3 (2001-2005), possibly relating to the discovery of the mechanism of anti-inflammatory and RA-induced cardiovascular diseases. Similar to P_1_, as shown in Figure 2D, the CI of RA peaked in 1956-1960 (40.44%), then declined to 1.02% in 2016-2018, indicating that around 40% of authors in Phase 1 were studying RA, but only about 1.02% authors still worked on the same disease in Phase 4.

In summary, in Phase 1, the P_1_, P_2_, P_3_, and CI of RA reached their maxima, or showed a significant increase, indicating that RA was the disease upon which most research was focused in the aspirin domain at this time. However, the value of these indicators showed profound declines in the next 3 phases, which means that aspirin was studied in relation to other diseases and is thus an ideal example of drug repurposing.

### Scientific Basis for Repurposing

**Figure 3:**
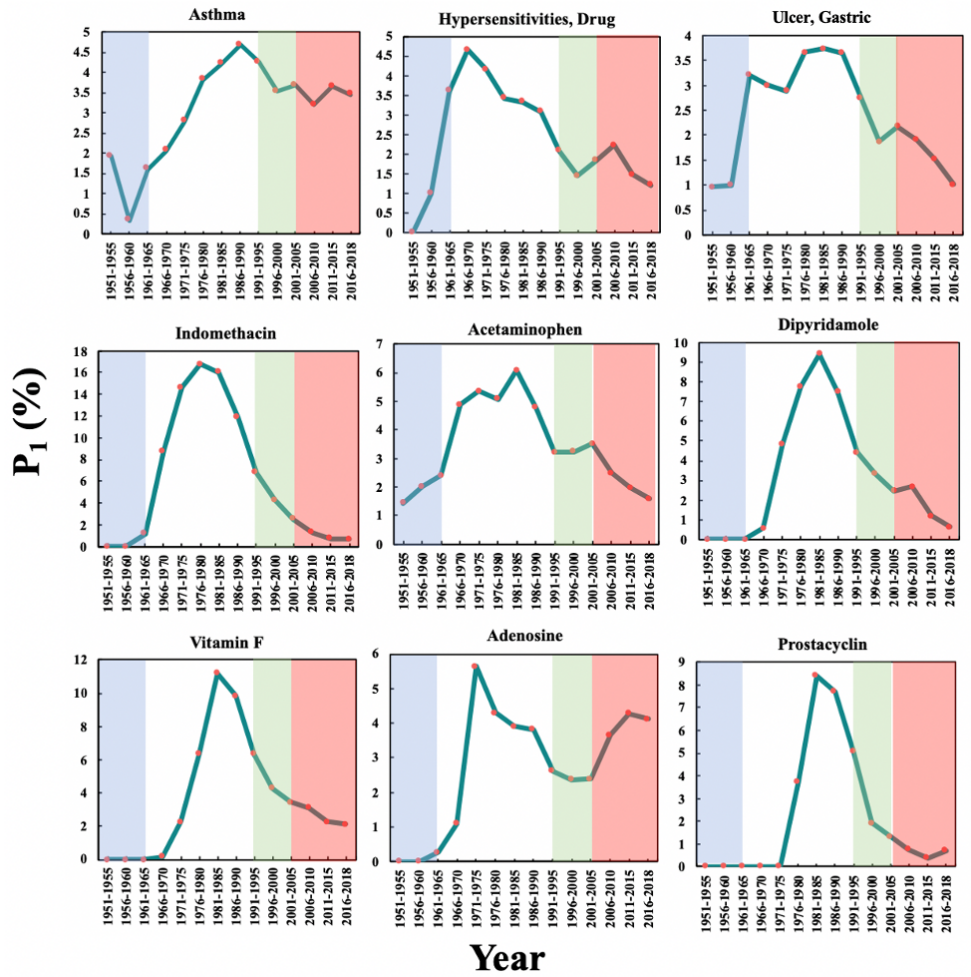
The Popularity Index (P_1_) of the biomedical entities on the pharmacological mechanisms and side effects of aspirin over time. The background colors show the 4 phases of aspirin research.

As shown in Figure 3, there are 9 top biomedical entities in the aspirin domain that reached their maximum P_1_ in Phase 2, including 3 diseases (“asthma”; “hypersensitivities, drug”; and “ulcer, gastric”) and 6 drugs (indomethacin, acetaminophen, dipyridamole, vitamin F, adenosine, and prostacyclin). The 3 diseases can all be side effects of aspirin, while the 6 drugs can be divided into 3 categories: (1) competitors of aspirin, that is, indomethacin and acetaminophen, which are analgesic and antipyretic drugs, respectively, with fewer side effects; (2) the antiplatelet drug dipyridamole; and (3) precursor substances in the pathway of the mechanism of action of aspirin (vitamin F, adenosine, and prostacyclin). In contrast with RA, the P_1_ of these biomedical entities increased from Phase 1, peaked in Phase 2, and then decreased, indicating that the side effects and mechanisms of aspirin were studied in detail in Phase 2. The P_1_ of indomethacin in 1976-1980 (16.75%) was the highest among these 9 entities in Phase 2, and vitamin F in 1981-1985 (11.19%) ranked second.

**Figure 4:**
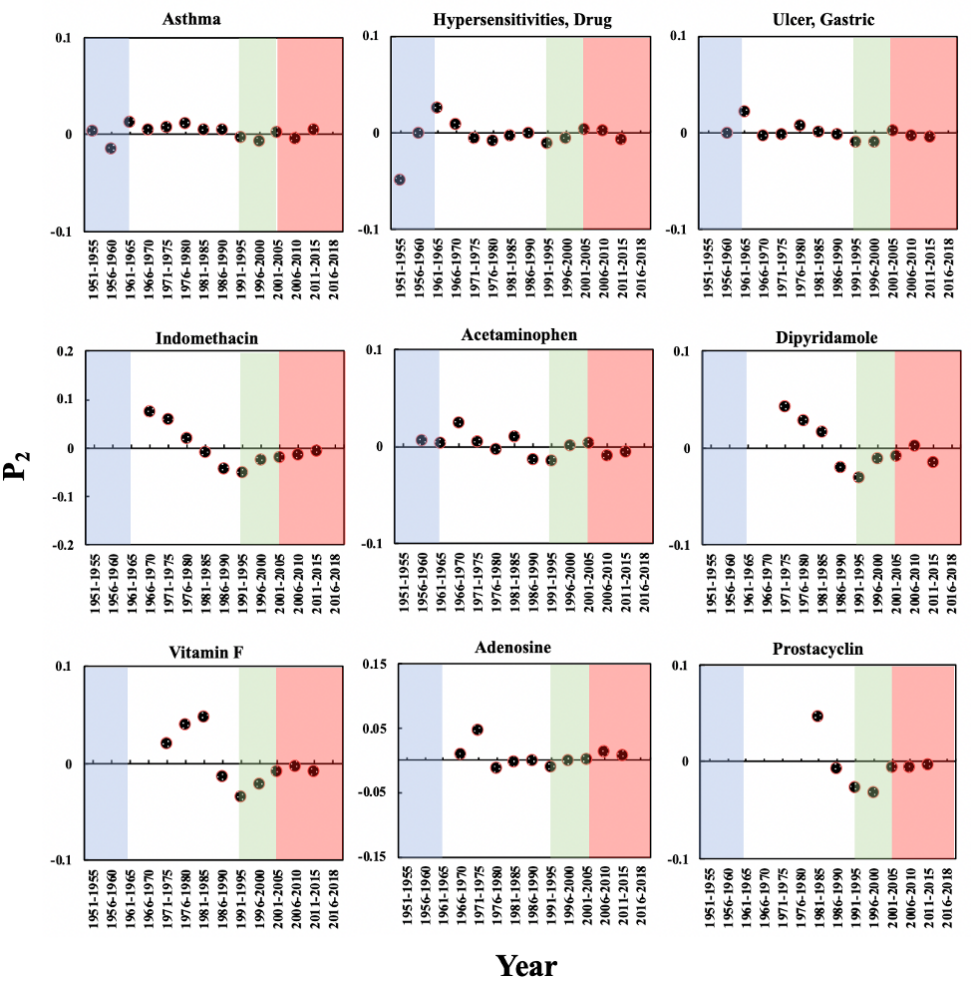
The Promising Index (P_2_) of the biomedical entities on the pharmacological mechanisms and side effects of aspirin over time. The background colors show the 4 phases of aspirin research.

Figure 4 shows the P_2_ of these 9 biomedical entities in the aspirin domain over time. The P_2_ of the 3 side effects had a significant increase of more than zero in Phase 2, indicating that interest in the side effects of aspirin increased sharply: 1961-1965 and 1976-1980, for “asthma”; 1961-1965 for “hypersensitivities, drug”; and 1961-1965 for “ulcer, gastric.” The time periods in which the P_2_ of the 6 drugs showed significant increases are generally later than those for the side effects, such as 1971-1975 for indomethacin and 1981-1985 for prostacyclin. This observation indicates that the discovery and in-depth study of side effects may have positive effects on the discovery of the mechanism of action of aspirin as well as the development of alternatives with fewer side effects.

**Figure 5:**
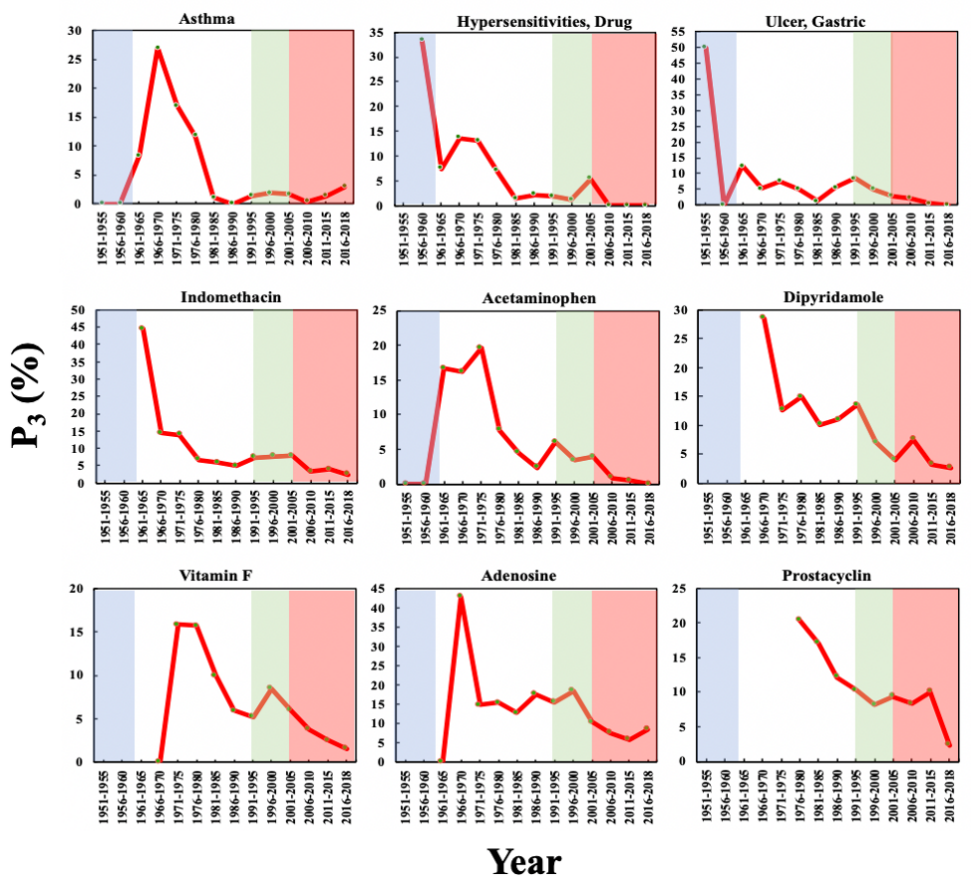
The Prestige Index (P_3_) of the biomedical entities on the pharmacological mechanisms and side effects of aspirin over time. The background colors show the 4 phases of aspirin research.

Figure 5 shows the P_3_ of these 9 biomedical entities in the aspirin domain, demonstrating a feature common to all 9 entities: a gradual decline with a fluctuation in P_3_ after reaching a maximum in Phase 1 or Phase 2. The highest initial P_3_ values of “hypersensitivities, drug” and “ulcer, gastric” occurred in Phase 1, revealing that both side effects had been taken seriously by researchers in Phase 1. The P_3_ of “hypersensitivities, drug” in 1956-1960 (33.33%) was higher than that of RA in 1956-1960 (25.00%). In 2011-2015, the P_3_ of only 2 entities are over the 5% threshold: 5.82% for adenosine and 10.00% for prostacyclin. In the aspirin domain, papers studying these 2 entities published in the top 20 journals comprised more than 5% of papers published in all of the journals indexed by the PubMed in 2011-2015. This observation indicates that the 2 entities were still important foci of research in the aspirin domain.

It can be observed from Figure 3, Figure 5, and Table 3 that P_3_, on average, achieved its maxima 10.7 years earlier than P_1_. In particular, for “hypersensitivities, drug” and “ulcer, gastric,” the intervals can be as long as 20 years. This observation indicates that P_3_ can reflect an early sign of academic interest into biomedical entities, a phenomenon that could be potentially valuable for tracking the research frontiers of a drug.

**Table 3:** Intervals between the time periods of the maxima of P_1_ and P_3_.

Biomedical entity	Time period of the maximum of P_1_ (T1)	Time period of the maximum of P_3_ (T2)	T1-T2 (years)
Asthma	1986-1990	1966-1970	20
Hypersensitivities, drug	1966-1970	1956-1960	10
Ulcer, gastric	1976-1980	1956-1960	20
Indomethacin	1976-1980	1961-1965	15
Acetaminophen	1981-1985	1971-1975	10
Dipyridamole	1981-1985	1966-1970	15
Vitamin F	1981-1985	1971-1975	10
Adenosine	1971-1975	1966-1970	5
Prostacyclin	1981-1985	1976-1980	5

**Figure 6:**
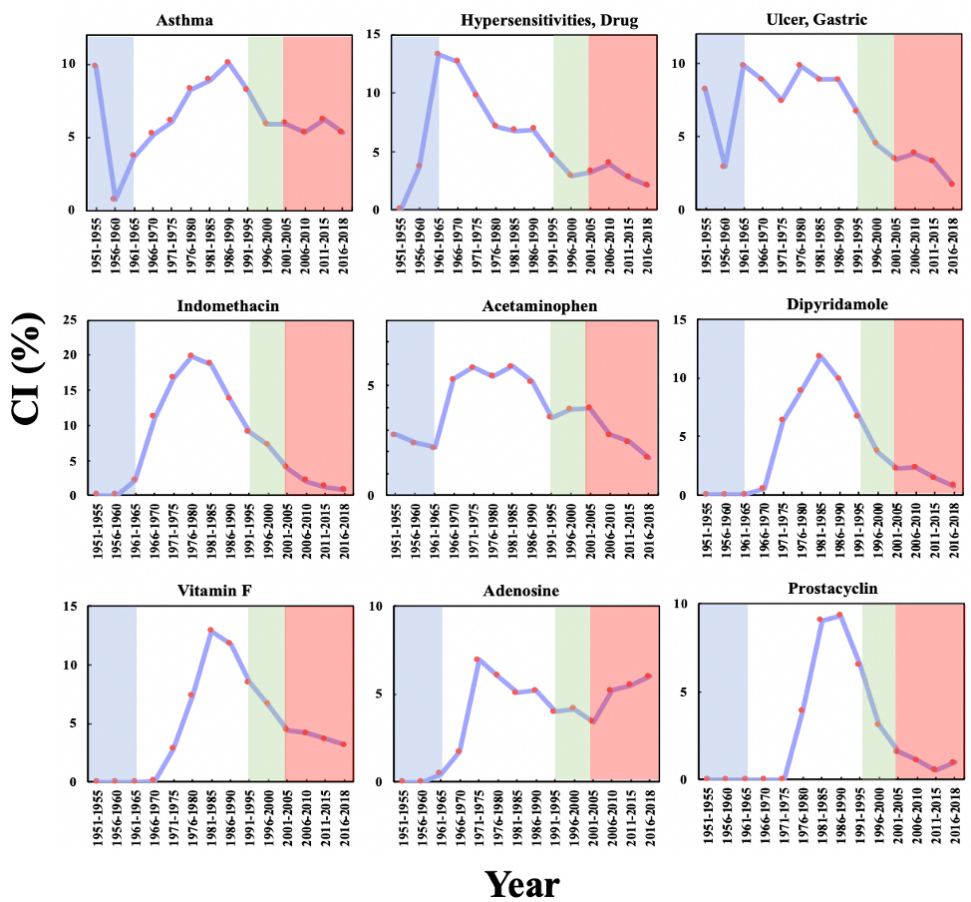
Collaboration Index (CI) of the biomedical entities on the pharmacological mechanisms and side effects of aspirin over time. The background colors show the 4 phases of aspirin research.

The results of the CI of these 9 biomedical entities in the aspirin domain are presented in Figure 6, which shows that the CIs for these biomedical entities have similar trends to those of P_1_ over time. Among all 9 biomedical entities during 1951-2018, indomethacin achieved the highest maximum CI in 1976-1980 (19.79%), indicating that it became a strong competitor to aspirin as an analgesic agent in Phase 2. This result also demonstrates that during the last 5-year period (2011-2015), the CIs of only 2 of the 9 entities were >5%, indicating that the 2 entities were still the subject of research by a considerable number of scientists (>2230) in the aspirin research community in 2011-2015. The 2 biomedical entities include “asthma” (6.21%) and adenosine (5.50%).

Based on the observation of P3C in Phase 2 and previous studies on aspirin [34,35], we can conclude that, on one hand, the in-depth investigation of the side effects and mechanism of action of aspirin provided the knowledge basis and research direction for drug repurposing. On the other hand, due to the market competition from other drugs, as well as the serious side effects, pharmaceutical companies attempted to discover new indicators for aspirin, in order to maintain the sales volume of aspirin.

### Repurposing Aspirin for Cardiovascular-Related Diseases

**Figure 7:**
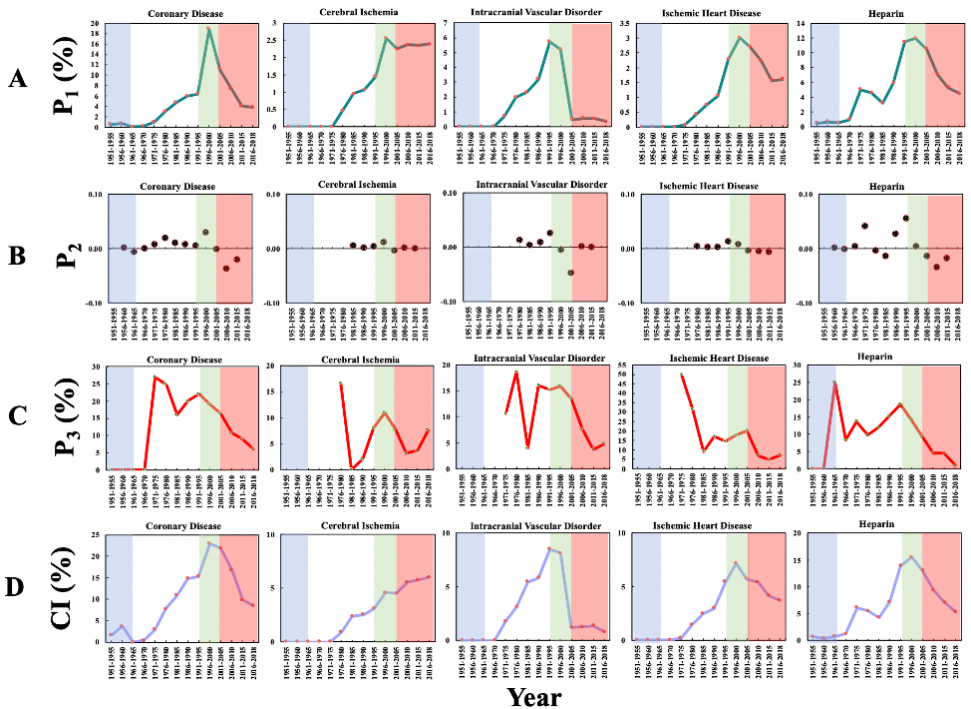
The 4 entitymetric indexes of the biomedical entities on cardiovascular diseases in the aspirin domain over time. The background colors show the 4 phases of aspirin research.

In Phase 3, 5 top biomedical entities comprising 4 diseases and 1 drug reached their maximum P_1_, as shown in Figure 7A. The 4 diseases were all cardiovascular-related, including “coronary disease” (P_1_ of 18.88% in 1996-2000), “cerebral ischemia” (P_1_ of 2.57% in 1996-2000), “intracranial vascular disorder” (P_1_ of 5.73% in 1991-1995), and “ischemic heart disease” (P_1_ of 3.01% in 1996-2000). Compared with Figures 2 and 3, the P_1_ of the previous 10 biomedical entities that peaked in the Phase 1 or Phase 2 were considerably lower than that of coronary disease, indicating that cardiovascular-related disease was the focus of the aspirin domain in that time. Coronary disease is often referred as ischemic heart disease and is the most common cardiovascular-related disease worldwide; similarly, cerebral ischemia and intracranial vascular disorder represent the same condition, commonly known as stroke. These conditions were reportedly the first and second most common causes of death worldwide in the early 21st century [74]. The demand for the prevention and treatment of such fatal diseases could be one of the factors driving the repurposing of aspirin for cardiovascular-related diseases.

The only drug that reached its maximum P_1_ in Phase 3 is heparin (11.92% in 1996-2000). As one of the most common anticoagulant drugs, heparin has always been the reference drug for repurposing aspirin to treat cardiovascular-related diseases, which could be the reason for the increase in the academic interest in heparin in the aspirin domain. There was another peak of heparin in Phase 2 (5.03%, 1971-1975), which could be related to an increase in research into the mechanisms of the antiplatelet effect of aspirin in Phase 2.

Figure 7B shows the changes in P_2_ of these 5 biomedical entities over time. All 5 biomedical entities demonstrated a significant increase in Phase 3. “Coronary disease” and “cerebral ischemia” increased in 1991-1995, and “intracranial vascular disorder”, “ischemic heart disease,” and heparin increased in 1991-1995. The P_2_ of the 2 entities also showed significant increases in Phase 2, consistent with the fact that aspirin was clinically used for coronary disease before the discovery of its antiplatelet effect: 0.02 in 1976-1980 for “coronary disease” and 0.10 in 1971-1975 for heparin.

The pattern of P_3_ for these 5 entities over time is displayed in Figure 7C. All 5 biomedical entities reached their maxima in Phase 2, earlier than the maximum of P_1_. “Coronary disease” reached a maximum in 1971-1975, and heparin reached a maximum in 1961-1965. The difference from the previous phases is that the P_3_ of these 5 biomedical entities peaked again in Phase 3. For instance, “coronary disease” peaked in 1991-1995, and heparin peaked in 1991-1995, indicating that these biomedical entities were important topics of research in both Phase 1 and Phase 3.

Figure 7D shows the CI of the 5 biomedical entities during 1951-2018, in which the CI demonstrated a dynamic trajectory very similar to that of P_1_. The maximum of “coronary disease” in Phase 3 is highest at 22.91% in 1996-2000, indicating that “coronary disease” attracted the greatest share of the authors in the aspirin domain. “Coronary disease” and “cerebral ischemia” in Phase 4 and heparin in Phases 2 and 4 surpassed the threshold value of 5%. The CI of “cerebral ischemia” steadily grew after Phase 3, showing a different trend from the other 4 biomedical entities, which increased in Phase 1 and Phase 2, peaked in Phase 3, and then dramatically decreased. This observation may illustrate that “cerebral ischemia,” unlike the other biomedical entities, is still increasing in popularity and collaboration, so additional increases are still expected.

### Repurposing Aspirin for Other Diseases

**Figure 8:**
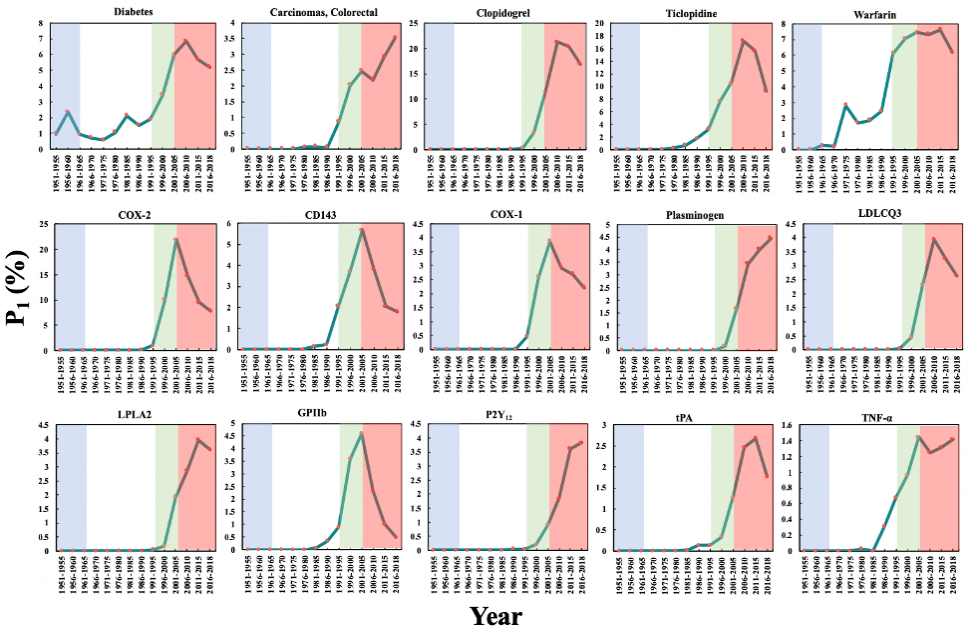
The Popularity Index (P_1_) of the biomedical entities on repurposing aspirin for other diseases over time. The background colors show the 4 phases of aspirin research.

In Figure 8, there are 15 biomedical entities that reached their maximum P_1_ in Phase 4. Unlike the previous phases, most of the biomedical entities were genes and can be divided into 3 categories according to the diseases to which they are related: (1) inflammatory-related genes (eg, COX-2, LPLA2, and TNF-α), (2) cardiovascular-related genes (eg, COX-1, CD143, plasminogen, LDLCQ3, GPIIb, P2Y_12_, and tPA), and (3) cancer-related genes (eg, TNFa, COX-2, COX-1, and LPLA2). These observations indicate that aspirin was actively studied for these 3 aspects of diseases from the perspective of genes in Phase 4. In particular, the maximum P_1_ of COX-2 was the highest among these 15 biomedical entities at 21.97% in 2001-2005, revealing that COX-2 was considered to be very important in the aspirin domain at that time.

Figure 8 also shows that the P_1_ of 2 diseases peaked in Phase 4. One is “diabetes,” whose P_1_ in 2006-2010 was 6.83%. In fact, as early as 1875, Ebstein and Müller [75] discovered that aspirin had the effect of lowering blood glucose levels. Inspired by this observation, scientists have since been trying to use aspirin for the treatment of diabetes [75]. There are several peaks in the P_1_ of “diabetes” in previous phases. In the 21st century, it has been recommended that patients with diabetes who have an increased risk of cardiovascular disease take aspirin as a primary preventative [5,76]; this could be the reason why the academic interest in “diabetes” in the aspirin domain increased again. The other disease is “carcinomas, colorectal.” Its P_1_ peaked in 2001-2005 and then increased significantly after a small decline in 2006-2010, a pattern which is very different from other diseases in the aspirin domain. Repurposing aspirin for the treatment of colorectal carcinomas appears to be a focus of research in the aspirin domain today. The P_1_ of 3 drugs also peaked in Phase 4, including the antiplatelet drugs clopidogrel and ticlopidine, which are competitors of aspirin as antiplatelet drugs [35], and warfarin, which is an anticoagulation drug that is similar to heparin and has been found to be superior to aspirin for secondary prevention of ischemic stroke with nonvalvular atrial fibrillation [77,78].

**Figure 9:**
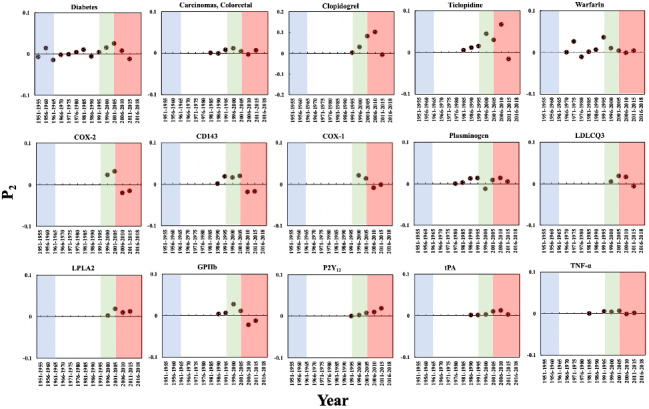
The Promising Index (P_2_) of the biomedical entities on repurposing aspirin for other diseases over time. The background colors show the 4 phases of aspirin research.

Figure 9 presents the changes in P_2_ of these 15 biomedical entities over time. All of the genes demonstrate an increase of more than 0 in Phase 4. Unlike these genes, the diseases and drugs showed several significant increases of more than 0 in different phases, which reflects a longer history of research in the aspirin domain. For example, the increases occurred in 1956-1960, 1996-2000, and 2001-2005 for “diabetes”; 1996-2000, 2001-2005, and 2006-2010 for clopidogrel; and 1971-1975 and 1991-1995 for warfarin.

**Figure 10:**
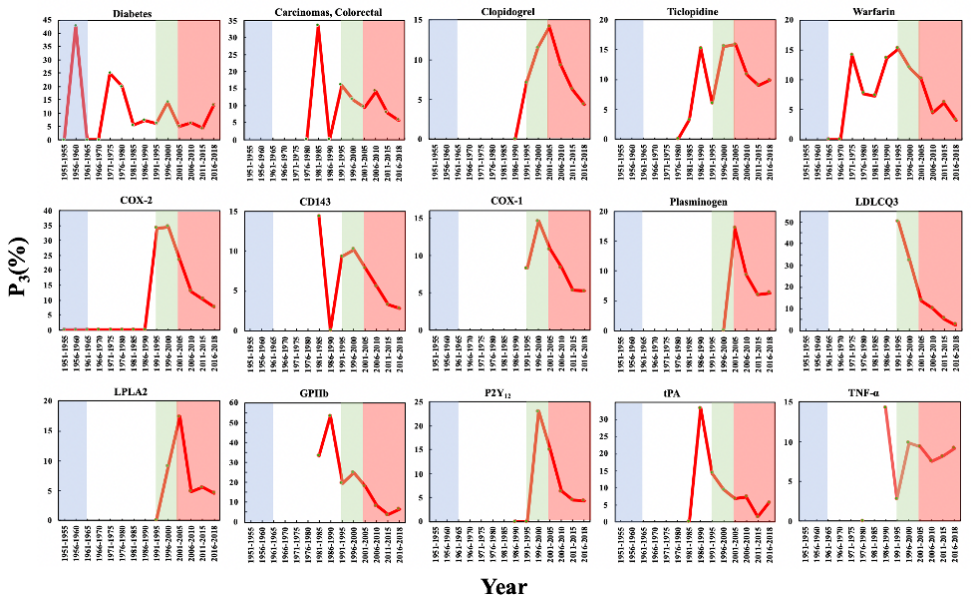
The Prestige Index (P_3_) of the biomedical entities on repurposing aspirin for other diseases over time. The background colors show the 4 phases of aspirin research.

The changes in P_3_ of these 15 biomedical entities over time are shown in Figure 10, from which we can make two observations. First, the P_3_ of these biomedical entities demonstrated that the time period of the maximum of P_3_ was much earlier than that of the maximum of P_1_. Second, unlike the biomedical entities noted in previous sections, the diseases and drugs had ≥2 significant peaks in different phases. For instance, “diabetes” had peaks of 42.86% in 1956-1960, 25.00% in 1971-1975, and 14.22% in 1996-2000, and “carcinomas, colorectal” had peaks of 33.33% in 1981-1985, 15.91% in 1991-1995, and 14.15% in 2006-2010. These numbers indicate that these entities attracted considerable interest in the field of aspirin research and high-impact papers on these conditions were published. However, the genes usually had only one peak in P_3_ in Phase 3 or 4, illustrating that these genes are relatively new topics in the aspirin domain.

**Figure 11:**
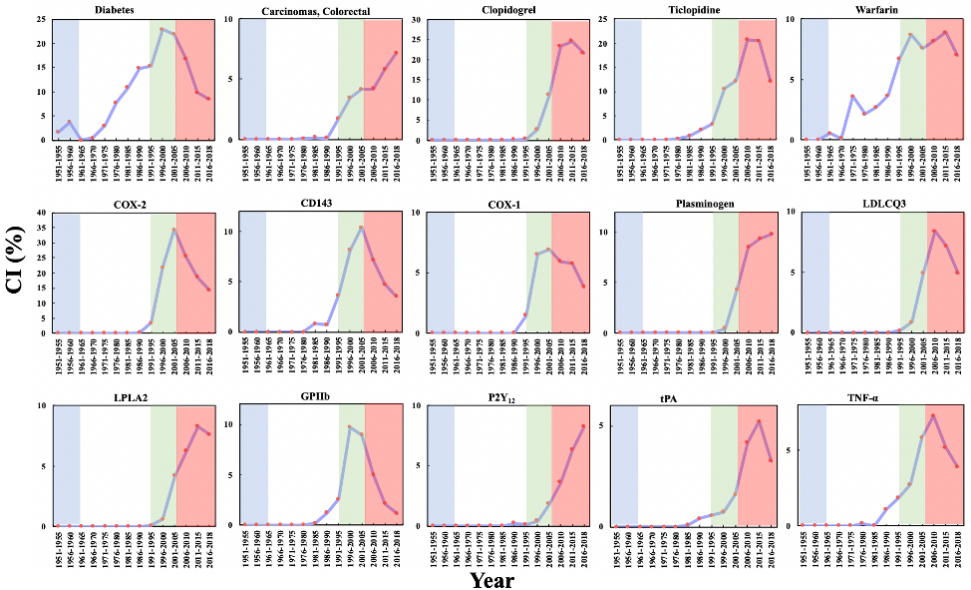
The Collaboration Index (CI) of the biomedical entities on repurposing aspirin for other diseases over time. The background colors show the 4 phases of aspirin research.

The CI data for these 15 biomedical entities are presented in Figure 11, which shows that the maximum CI for COX-2 is the highest, at 34.37%, in 2001-2015, denoting that COX-2 was the focus of aspirin research in Phase 4; the research and development of Vioxx, a selective COX-2 inhibitor with fewer side effects, may be one of the reasons [79]. The CI of 2 drugs, clopidogrel (25.54% in 2001-2015) and ticlopidine (20.74% in 2006-2010), reveals fierce competition between aspirin and these alternative antiplatelet drugs. This competition could have driven the repurposing of aspirin for other diseases, especially cancers, that have an urgent demand for effective treatment.

## Discussion

### Principal Findings

This study examines drug repurposing from the perspective of the evolution of biomedical entities, using aspirin as the study subject. It is of paramount importance for drug discovery to identify the factors that drive repurposing as well as potential patterns among biomedical entities in various phases of the drug research timeline. The main contribution of this paper is twofold. First, we proposed 4 entitymetric indices (P3C) to quantify changes in academic interest in biomedical entities and to reveal the granular process of drug repurposing. Second, we divided aspirin research into 4 phases, including original use (1951-1960), in-depth studies of pharmacological mechanisms and side effects (1961-1990), repurposing for cardiovascular-related diseases (1991-2000), and repurposing for other diseases (2001-2018), taking into consideration 3 granular perspectives—disease, drug, and gene—that contribute to a comprehensive understanding of the features of the repurposing process.

Our entitymetric results indicate that aspirin is representative of the process of drug repurposing. The research findings can be summarized as follows. In Phase 1, aspirin was routinely used to ease pain, fever, and inflammation and was often used in the treatment of RA [34], with a P3C that peaked in 1951-1960. Despite the widespread use of aspirin, at this stage, its mechanism of action was not well understood [34]. In Phase 2, the side effects and mechanisms of actions of aspirin were studied extensively, as shown by the maxima of P_1_ and CI, as well as a significant increase in P_2_ for the relevant biomedical entities in 1961-1990. The anti-platelet effect [70], inhibition of prostaglandin synthesis [71], and acetylation effect on the enzyme cyclo-oxygenase [72] were uncovered. These discoveries provided a solid knowledge foundation for the successful repurposing of aspirin. The highest P_1_ in 1961-1990 was for indomethacin (16.75%), denoting fierce competition with aspirin for its original use. This could be one of the factors contributing to the repurposing of aspirin.

In Phase 3, aspirin was successfully used for several cardiovascular-related diseases because of its antiplatelet effects [80]. The related diseases and drugs achieved their highest values of P_1_ and CI as well as significant increases in P_2_ in 1991-2000. As these diseases are the most common diseases worldwide, according to data from the World Health Organization [74], the demand for the prevention and treatment of fatal diseases is also another potential factor driving drug repurposing. In the last phase, there was a large number of studies suggesting the use of aspirin for other diseases, especially colorectal cancer [36]. The greatest difference from previous phases is that aspirin was studied at the genetic level. Ten genes reached their maxima of P_1_ and CI as well as an apparent increase in P_2_ in 2001-2018. This observation could indicate that the development of modern science and technology, such as gene sequencing, molecular simulation, and deep learning, accelerates the process of drug repurposing of aspirin. Meanwhile, 2 fatal diseases — diabetes and colorectal carcinoma — as well as 3 competitive drugs of aspirin as an antiplatelet agent — clopidogrel, ticlopidine, and warfarin (an anticoagulant and competitor with aspirin for stroke prevention) — also had peak P_1_ and CI values and a great increase in P_2_.

Methodologically, in this study, we developed 4 entitymetrics and demonstrated how to use them to investigate the process of drug repurposing. The results demonstrate that the maxima of P_1_, P_3_, and CI are closely associated with the different phases of research of aspirin repurposing. The P_1_ and CI metrics can indicate dynamic trends in academic interest in a given biomedical entity over a long time period. For instance, long-lasting increases in P_1_ and CI signal interest in repurposing, while P_2_ is more sensitive to immediate changes in academic interest in a specific biomedical entity, since it takes into consideration data from the two most recent periods. Moreover, P_3_ can reflect a research focus far earlier than the other 3 indices, which means that a continuously high P_3_ may be valuable as an early signal of the emergence and transfer of research topics in drug research. If P_3_ does indeed have predictive power, it could be due to the involvement of top domain experts in the peer review of manuscripts in top journals with high impact factors [81,82]. Additionally, due to their easy implementation and interpretability, these indices can be applied in multiple domains, such as drug assessment, drug discovery, and pharmacovigilance.

### Limitations and Future Directions

There are several limitations in the current paper. First, the data included in our analysis are limited to articles indexed in PubMed. Some real-world data, such as electronic health records, clinical trials, and social media, in which aspirin and its related biomedical entities were mentioned, should be included. In our future work, we will use different types of data sources for studying drug repurposing and take into account other entities related to drugs, including other biomedical entities, such as pathways, proteins, and cells, and non-biomedical entities, such as authors, institutions, and countries. The landscape of collaborations between academic institutions and pharmaceutical companies could affect the drug repurposing process. Second, there are several ways of measuring the impact of a journal, such as the impact factor and relative citation ratio. Third, this study mainly focused on investigating the repurposing journey of aspirin, but we did not test whether it can be used to predict future drug repurposing. In future studies, we will evaluate the different impact measures of a journal and choose a proper measure better fitted to the chosen drug. Furthermore, we will also aim to test the proposed metrics on other drugs to understand their repurposing journeys (eg, metformin) to see whether generalized patterns exist in different repurposing processes.

## Appendix

Multimedia Appendix 1 Top 20 journals related to aspirin research.

Multimedia Appendix 2 Changes in the number of journals with aspirin-related publications during 1951-2018. The top 5 journals and their frequencies are indicated using heat maps for every 5-year period.

Multimedia Appendix 3 Table S2 summarizes the peaks of P_1_, P_3_, and CI as well as the increase in P_2_ for all the top 30 bioentities. The details can be found in the supplementary information section, including Phase 2 (1961-1990, the scientific basis for repurposing), Phase 3 (1991-2000, repurposing aspirin for cardiovascular-related diseases), and Phase 4 (2001-2018, repurposing aspirin for other diseases).

## Abbreviations

CI: Collaboration Index
DR: drug repurposing
GWAS: genome-wide association study
P_1_: Popularity Index
P_2_: Promising Index
P_3_: Prestige Index
P3C: the 4 entitymetric indicators for biomedical entities
RA: rheumatoid arthritis
