# Supplementary material for: Understanding Drug Repurposing From the Perspective of Biomedical Entities and Their Evolution: Bibliographic Research Using Aspirin
Source: JMIR Med Inform. 2020 Jun 16;8(6):e16739. doi: 10.2196/16739 (PMC7327595; doi:10.2196/16739)
Supplement: Multimedia Appendix 1 [file medinform_v8i6e16739_app1.docx]

Table S1 Top 20 journals related to aspirin research.

| **NO.** | **Name** |
| --- | --- |
| 1 | THE NEW ENGLAND JOURNAL OF MEDICINE |
| 2 | LANCET |
| 3 | JAMA |
| 4 | BRITISH MEDICINE JOURNAL |
| 5 | NATURE |
| 6 | NATURE REVIVEW. DRUG DISCOVERY |
| 7 | NATURE MEDICINE |
| 8 | SCIENCE |
| 9 | PHARMACOLOGICAL REVIEWS |
| 10 | ANNALS OF INTERNAL MEDICINE |
| 11 | THE JOURNAL OF CLINICAL INVESTIGATION |
| 12 | PROCEEDINGS OF THE NATIONAL ACADEMY OF SCIENCE OF THE UNITED STATES OF AMERICA |
| 13 | TRENDS IN THE PHARMACOLOGICAL SCIENCES |
| 14 | THE JOURNAL OF PHARMACOLOGY AND EXPERIMENTAL THRAPEUTICS. |
| 15 | CIRCULATION |
| 16 | JOURNAL OF THE AMERICAN COLLEGE OF CARDIOLOGY |
| 17 | BRITISH JOURNAL OF HAEMATOLOGY |
| 18 | BLOOD |
| 19 | EUROPEAN HEART JOURNAL |
| 20 | JOURNAL OF THROMBOSIS AND HAEMOSTASIS |
