# Supplementary material for: Understanding Drug Repurposing From the Perspective of Biomedical Entities and Their Evolution: Bibliographic Research Using Aspirin
Source: JMIR Med Inform. 2020 Jun 16;8(6):e16739. doi: 10.2196/16739 (PMC7327595; doi:10.2196/16739)
Supplement: Multimedia Appendix 2 [file medinform_v8i6e16739_app2.docx]

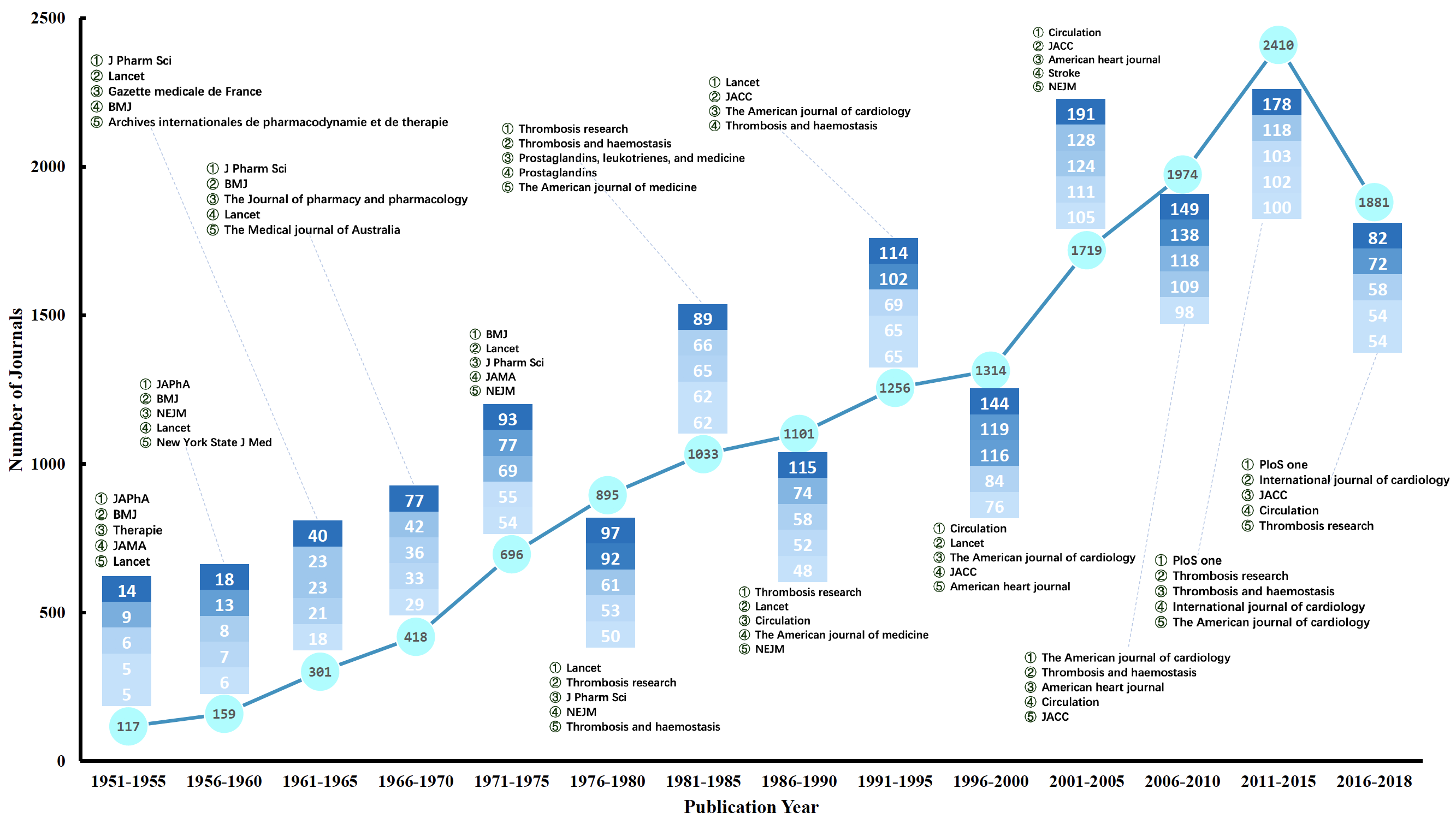


Figure S1 Changes in the number of journals with aspirin-related publications during 1951-2018. The top five journals and their frequencies are indicated using heatmaps for every five-year period.
