# Supplementary material for: Understanding Drug Repurposing From the Perspective of Biomedical Entities and Their Evolution: Bibliographic Research Using Aspirin
Source: JMIR Med Inform. 2020 Jun 16;8(6):e16739. doi: 10.2196/16739 (PMC7327595; doi:10.2196/16739)
Supplement: Multimedia Appendix 3 [file medinform_v8i6e16739_app3.docx]

Table S2 The entitymetric results for the top 30 bio-entities

| **Time Period**  **Entity Name** | **Phase 1 (1951-1960)** | | | | **Phase 2 (1961-1990)** | | | | **Phase 3 (1991-2000)** | | | | **Phase 4 (2001-2018)** | | | |
| --- | --- | --- | --- | --- | --- | --- | --- | --- | --- | --- | --- | --- | --- | --- | --- | --- |
|  | ***P_1_ (%)*** | ***P_2_ > 0*** | ***P_3_ (%)*** | ***CI (%)*** | ***P_1_ (%)*** | ***P_2_ > 0*** | ***P_3_ (%)*** | ***CI (%)*** | ***P_1_ (%)*** | ***P_2_ > 0*** | ***P_3_ (%)*** | ***CI (%)*** | ***P_1_ (%)*** | ***P_2_ > 0*** | ***P_3_ (%)*** | ***CI (%)*** |
| coronary disease |  |  |  |  |  | (71-90) | 26.92  (71-75) |  | 18.88  (96-00) | (96-00) | 22.02  (91-95) | 22.91  (96-00) |  |  |  |  |
| asthma |  |  |  | 9.84  (51-5`5) | 4.68  (86-90) | (61-65)  (71-80) | 26.92  (66-70) | 10.08  (86-90) |  |  | 1.79  (96-00) |  |  |  |  |  |
| diabetes | 2.34  (56-60) | (56-60) | 42.86  (56-60) | 3.68  (56-60) | 2.12  (81-85) | (81-85) | 25.00  (71-75) |  |  | (96-00) | 14.22  (96-00) | 22.91  (96-00) | 6.83 (06-10) | (01-10) |  |  |
| hypersensitivities, drug |  |  | 33.33  (56-60) |  | 4.65  (66-70) | (61-70) | 13.56  (66-70) | 13.28  (61-65) |  |  |  |  |  |  | 5.37  (01-05) |  |
| ulcer, gastric |  |  | 50  (51-55) | 9.85  (61-65) | 3.73  (81-85) | (61-65)  (76-80) | 12.5  (61-65) |  |  |  | 8.45  (91-95) |  |  |  |  |  |
| cerebral ischemia |  |  |  |  |  |  | 16.67  (76-80) |  | 2.57 (96-00) | (96-00) | 11.04  (96-00) |  |  |  |  | 5.99  (16-18) |
| intracranial vascular disorder |  |  |  |  |  |  | 18.67  (76-80) |  | 5.73 (91-95) | (91-95) | 15.96  (96-00) | 8.50  (91-95) |  |  |  |  |
| ischemic heart disease |  |  |  |  |  |  | 50  (71-75) |  | 3.01 (96-00) | (91-00) |  | 7.14  (96-00) |  |  | 20.18  (01-05) |  |
| carcinomas, colorectal |  |  |  |  |  |  | 33.33  (81-85) |  |  | (91-00) | 15.91  (91-95) |  | 3.52 (16-18) |  | 14.15  (06-10) | 7.15  (16-18) |
| rheumatoid arthritis | 9.36 (56-60) | (51-55) | 25  (56-60) | 40.44  (56-60) |  |  | 16.57  (71-75) |  |  |  |  |  |  |  | 5.05  (01-05) |  |
| clopidogrel |  |  |  |  |  |  |  |  |  | (96-00) |  |  | 21.32 (06-10) | (01-10) | 14.13  (01-05) | 24.54  (11-15) |
| ticlopidine |  |  |  |  |  | (86-90) | 15.19  (86-90) |  |  | (91-00) |  |  | 17.24 (06-10) | (01-10) | 15.83  (01-05) | 20.74  (06-10) |
| heparin |  |  | 25  (61-65) |  |  | (71-75)  (86-90) | 13.67  (71-75) | 6.08  (71-75) | 11.92 (96-00) | (91-95) | 18.58  (91-95) | 15.50  (96-00) |  |  |  |  |
| indomethacin |  |  | 44.44  (61-65) |  | 16.75 (76-80) | (66-80) |  | 19.79  (76-80) |  |  |  |  |  |  |  |  |
| warfarin |  |  |  |  |  | (71-75)  (86-90) | 14.10  (71-75) | 3.59  (71-75) |  | (91-00) | 15.23  (91-95) | 8.69  (96-00) | 7.62 (11-15) |  | 6.16  (11-15) | 8.89  (11-15) |
| vitamin f |  |  | 15.87  (71-75) |  | 11.19 (81-85) | (76-85) |  | 12.90  (81-85) |  |  | 8.46  (96-00) |  |  |  |  |  |
| dipyridamole |  |  |  |  | 9.42 (81-85) | (76-85) | 28.57  (66-70) | 11.80  (81-85) |  |  | 13.54  (91-95) |  |  |  | 7.6  (06-10) |  |
| adenosine |  |  |  |  | 5.64  (71-75) | (71-75) | 42.85  (66-70) | 6.95  (71-75) |  |  | 18.54  (96-00) |  |  | (06-15) |  |  |
| acetaminophen |  |  |  |  | 6.07  (81-85) | (61-70) | 19.59  (71-75) | 5.90  (81-85) |  |  | 6.02  (91-95) |  | 3.51  (01-05) |  | 3.87  (01-05) |  |
| prostacyclin |  |  |  |  | 8.42  (81-85) | (81-85) | 20.42  (76-80) | 9.32  (86-90) |  |  |  |  |  |  | 10  (11-15) |  |
| COX-2 |  |  |  |  |  |  |  |  |  | (96-00) | 34.58  (96-00) |  | 21.97  (01-05) | (01-05) |  | 34.37  (01-05) |
| CD143 |  |  |  |  |  |  |  |  |  | (91-00) | 10.17  (96-00) |  | 5.68  (01-05) | (01-05) |  | 10.36  (01-05) |
| COX-1 |  |  |  |  |  |  |  |  |  | (96-00) | 14.63  (96-00) |  | 3.86  (01-05) | (01-05) |  | 6.88  (01-05) |
| Plasminogen |  |  |  |  |  | (86-90) |  |  |  | (91-95) |  |  | 4.45  (16-18) | (01-10) | 17.16  (01-05) | 9.79  (16-18) |
| LDLCQ3 |  |  |  |  |  |  |  |  |  |  | 50  (91-95) |  | 3.93  (06-10) | (01-10) |  | 8.38  (06-10) |
| LPLA2 |  |  |  |  |  |  |  |  |  |  |  |  | 3.97  (11-15) | (01-15) | 17.41  (01-05) | 8.27  (11-15) |
| GPIIb |  |  |  |  |  |  | 53.33  (86-90) |  |  | (91-00) | 25  (96-00) | 9.72  (96-00) | 4.59  (01-05) | (01-05) |  |  |
| P2Y_12_ |  |  |  |  |  |  |  |  |  |  | 23.07  (96-00) |  | 3.84  (16-18) | (01-15) |  | 8.17  (16-18) |
| tPA |  |  |  |  |  |  | 33.33  (86-90) |  |  |  |  |  | 2.68  (11-15) | (01-10) | 7.36  (06-10) | 5.22  (11-15) |
| TNF-α |  |  |  |  |  |  | 14.28  (86-90) |  | 1.44  (01-05) |  | 9.84  (96-00) |  |  | (01-05) |  | 7.27  (06-10) |

Table S2 summarizes the peaks of P_1_, P_3_, and CI as well as the increase in P_2_ for all the top 30 bioentities. The details can be found in the supplementary information section, including Phase 2 (1961-1990, the scientific basis for repurposing), Phase 3 (1991-2000, repurposing aspirin for cardiovascular-related diseases), and Phase 4 (2001-2018, repurposing aspirin for other diseases).
